# The curcumin analog EF24 is a novel senolytic agent

**DOI:** 10.18632/aging.101787

**Published:** 2019-01-28

**Authors:** Wen Li, Yonghan He, Rongping Zhang, Guangrong Zheng, Daohong Zhou

**Affiliations:** 1 School of Pharmaceutical Science and Yunnan Key Laboratory of Pharmacology for Natural Products, Kunming Medical University, Kunming, Yunnan 650500, China; 2 Department of Pharmacodynamics, College of Pharmacy, University of Florida, Gainesville, FL 32610, USA; 3 Department of Endocrinology, The Third People’s Hospital of Yunnan Province, Kunming, Yunnan 650011, China; 4 Department of Medicinal Chemistry, College of Pharmacy, University of Florida, Gainesville, FL 32610, USA

**Keywords:** cellular senescence, aging, curcumin, EF24, senolytic agent

## Abstract

Cellular senescence is a hallmark of aging because senescent cells (SCs) accumulate with aging and play a causative role in age-related diseases. Selectively eliminating SCs has been emerging as a new strategy for treating age-related diseases and extending healthspan. Curcumin and its analogs have some anti-aging activities. However, the mechanisms of their action have not been fully elucidated. In the present study, we investigated whether various curcumin analogs can function as a senolytic agent. The results from our studies show that among these curcumin analogs EF24 is the most potent and broad-spectrum senolytic agent. Mechanistically, EF24 selectively kills SCs by inducing SC apoptosis in a reactive oxygen species (ROS) production independent manner but associated with an increase in the proteasome degradation of the Bcl-2 anti-apoptotic protein family proteins known to play an important role in protecting SCs from apoptosis. In addition, EF24 can synergistically kill SCs with ABT-263, a Bcl-2 and Bcl-xl inhibitor and a known senolytic agent. These findings provide new insights into the mechanisms by which curcumin analogs function as an anti-aging agent and suggest that the curcumin analog EF24 has the potential to be used as a novel senolytic agent for the treatment of age-related diseases.

## Introduction

Cellular senescence occurs when normal cells stop to divide after extensive replication or exposure to stress. It has been considered as a hallmark of aging because senescent cells (SCs) accumulate in various tissues with aging [1]. The mechanisms by which SCs accumulate with aging have not been fully understood but may be attributable in part to immune senescence that decreases the ability of the body to clear SCs [2]. Although cellular senescence is a tumor-suppressive mechanism, SCs can play a causative role in aging and age-related diseases when they accumulate [3–5]. This suggestion is supported by the finding that genetic elimination of SCs in naturally aged mice through a transgene can delay various age-dependent deterioration in tissues and organs and extend their lifespan [6,7]. This seminal finding stimulates research to identify small molecules termed senolytic agents that can selectively kill SCs as potential therapeutics for age-related diseases. To date, several classes of senolytic agents have been identified, and most of them are natural compounds such as quercetin, fisetin, and piperlongumine [8–10]. Because natural senolytic compounds have the advantages of low toxicity, they may have a better chance to be translated into clinic to treat age-related diseases or can be used as a lead for the development of more specific and potent senolytic agents [11].

Curcumin, a natural compound extracted from the turmeric, has a broad range of biological and pharmacological activities, including antioxidant [12], anti-inflammatory [13], antimicrobial [14], and anti-cancer [15] activities. Numerous studies suggest that curcumin has some health benefits in delaying aging and may be useful in preventing and treating age-related diseases [16–18]. For example, curcumin was shown to prolong lifespan and extend healthspan in Drosophila melanogaster (fruit fly) [19] and Caenorhabditis elegans [20]. However, the clinical administration of curcumin is difficult due to its low aqueous solubility, poor oral bioavailability, and rapid degradation under physiological conditions [21]. To improve the bioavailability and biological activity of curcumin, many curcumin analogs were developed, including EF24 [22], HO-3867 [23], 2-HBA [24] and dimethoxycurcumin [25], which have been demonstrated to be more active than curcumin in preventing and treating various diseases and reducing age-dependent deterioration (such as cancer, inflammation, et al.). However, the mechanisms of their anti-aging action have not been fully elucidated.

Since pharmacological clearance of SCs has the potential to postpone age-related pathologies and increase healthy lifespan, we hypothesized that curcumin and its analogs may increase healthspan in part by functioning as novel senolytic agents. Therefore, in this study, we examined the senolytic activity of several curcumin analogs and found that EF24 is a novel potent and broad-spectrum senolytic agent. We show that EF24 can selectively reduce the viability of human SCs from different tissue origins and induced by different stresses. Its senolytic effect is likely attributable to the induction of apoptosis via proteasome-mediated downregulation of the Bcl-2 anti-apoptotic family proteins such as Bcl-xl. These findings provide new insights into the mechanisms by which curcumin and its analogs function as anti-aging agents and suggest that the curcumin analog EF24 has the potential to be used as a novel senolytic agent for the treatment of age-related diseases.

## RESULTS

### EF24 is the most potent senolytic agent among different curcumin analogs studied

**Figure 1:**
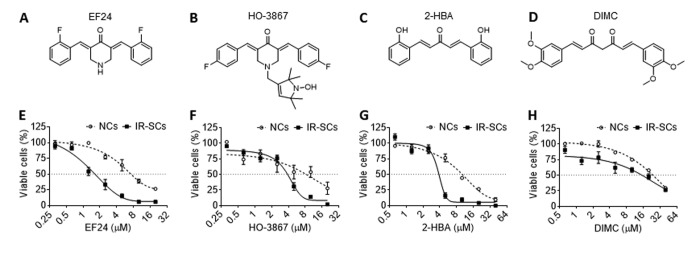
**Senolytic activities of different curcumin analogs.** (**A-D**) Chemical structures of EF24 (**A**), HO-3687 (**B**), 2-HBA (**C**) and dimethoxycurcumin (DIMC) (**D**). (**E-H**) Effect of EF24 (**E**), HO-3687 (**F**), 2-HBA (**G**) and DIMC (**H**) on the viability of WI-38 non-senescent cells (NCs) and IR‐induced senescent cells (IR-SCs) after the cells were treated by indicated concentrations of compounds for 72 h. Data are represented as mean ± SEM of three independent assays.

In this study, we first screened the senolytic activity of 4 commonly used curcumin analogs, i.e. EF24 (Figure 1A), HO-3867 (Figure 1B), 2-HBA (Figure 1C) and dimethoxycurcumin (DIMC) (Figure 1D), by measuring their effects on the viability of human WI-38 fibroblast non-senescent cells (NCs) and ionizing radiation induced SCs (IR-SCs). We found that EF24, HO-3867 and 2-HBA dose-dependently reduced the viability of IR-SCs but exhibited lesser toxicities to NCs (Figure 1E-G), whereas DIMC exhibited minimal selective toxicity against NCs and IR-SCs (Figure 1H). Among these analogs, EF24 is the most potent senolytic agent with an EC_50_ value of 1.62 μM compared to HO-3867 (EC_50_=3.85 μM), 2-HBA (EC_50_=3.85 μM) and DIMC (EC_50_=15.18 μM) against IR-SCs (Figure 1E-H; Table 1). In NCs, the EC_50_ values of EF24, HO-3867, 2-HBA and DIMC are 4.69 μM, 8.3 μM, 9.5 μM and 23.75 μM, respectively (Table 1). The EC_50_ value ratios of NCs and IR-SCs for EF24 is 2.9, these for HO-3867, 2-HBA and DIMC are 2.16, 2.47 and 1.56, respectively (Table 1). These results suggest that EF24 is the most potent and selective senolytic agent among these analogs.

**Table 1:** EC_50_ values of selective curcumin analogs against WI‐38 non-senescent cells (NCs) and IR-induced senescent cells (IR-SCs).

**Analogs**	**EC_50_ (µM)**			**EC_50_ value ratio**
	**NCs**	**IR-SCs**		**NCs/SCs**
EF24	4.69	1.62		2.90
HO-3867	8.30	3.85		2.16
2-HBA	9.50	3.85		2.47
DIMC	23.75	15.18		1.56

### EF24 is a broad-spectrum senolytic agent

**Figure 2:**
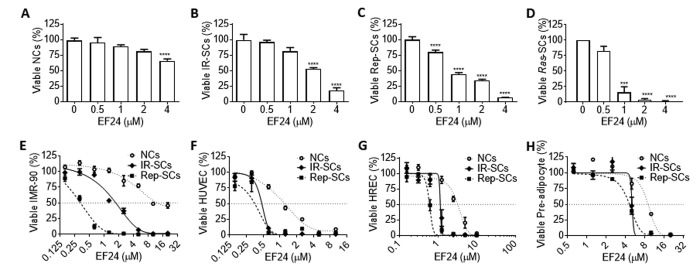
**EF24 is a broad-spectrum senolytic agent.** (**A-D**) Effect of EF24 on the viability of WI-38 non-senescent cells (NCs) (**A**), IR‐induced senescent cells (IR-SCs) (**B**), replication‐exhausted senescent cells (Rep‐SCs) (**C**), and Ras‐induced senescent cells (Ras‐SCs) (**D**) after the cells were treated with indicated concentrations of EF24 for 72 h. (**E-H**) Effect of EF24 on NCs, IR-SCs and Rep-SCs derived from IMR-90 fibroblasts (IMR-90) (**E**), human umbilical vein endothelial cells (HUVEC) (**F**), human renal epithelial cells (HREC) (**G**) and human pre-adipocytes (**H**) after they were treated with indicated concentrations of EF24 for 72 h. Data are represented as mean ± SEM of three independent assays. ***P < 0.001 and ****P < 0.0001 vs. vehicle control cells.

To rule out that the senolytic activity of EF24 is specific to WI-38 IR-SCs, we examined the effect of EF24 on the viability of WI-38 SCs induced by extensive replication (Rep-SCs) or ectopic expression of the *Ras* oncogene (*Ras*-SCs) along with IR-SCs and NCs. As showed in Figure 2A, EF24 had minimal effect on the cell viability of WI-38 NCs below 4 μM, while reduced the cell viability of WI-38 IR-SCs (Figure 2B), Rep-SCs (Figure 2C), and *Ras*-SCs (Figure 2D) in a dose-dependent manner at the same concentration range. Among these cells, *Ras*-SCs exhibited the highest sensitivity to EF24, followed by Rep-SCs and IR-SCs (Figure 2B-D). To explore whether the effect of EF24 is cell-type specific or EF24 has a broad-spectrum senolytic activity against different types of SCs, we measured the survival rates of NCs, IR-SCs and Rep-SCs from human IMR-90 fibroblasts (IMR-90, Figure 2E), human umbilical vein endothelial cells (HUVEC, Figure 2F), human renal epithelial cells (HREC, Figure 2G) and human pre-adipocytes (Figure 2H) after they were incubated with increasing concentrations of EF24. The results showed that EF24 reduced cell viability in all of these different types of cells when they became senescent in a dose-dependent manner, whereas NCs from all these tissue origins were more resistant to EF24 than SCs (Figure 2E-H). The summarized EC_50_ values and the ratios of NC and SC EC_50_ values for each of these cells are presented in Table 2, which demonstrates that EF24 is a broad-spectrum senolytic agent.

**Table 2:** EC_50_ values of EF24 against non-senescent cells (NCs), and IR- or Rep-induced senescent WI‐38 and IMR-90 fibroblasts, HUVEC, HREC and pre-adipocytes.

**Cell type**	**EC_50_ (μM)**				**EC_50_ value ratio**	
	**NCs**	**IR-SCs**	**Rep-SCs**		**NCs/IR-SCs**	**NCs/Rep-SCs**
WI-38	4.32	1.74	1.26		2.48	3.43
IMR-90	10.88	1.72	0.33		6.33	32.97
HUVEC	1.39	2.09	0.77		0.67	1.80
HREC	3.25	1.15	0.60		2.83	5.37
Pre-adipocyte	8.92	5.04	4.60		1.77	1.94

### EF24 induces senescent cell apoptosis

**Figure 3:**
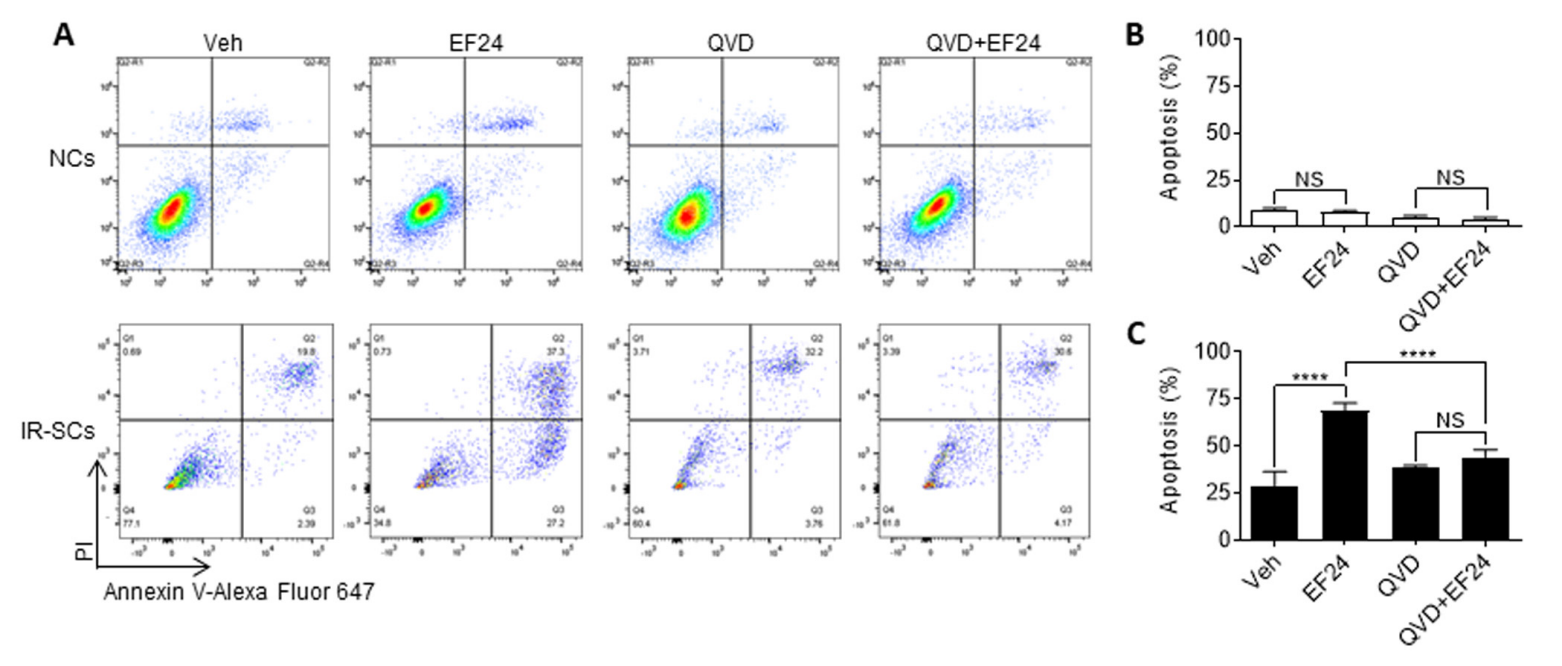
**EF24 selectively induces apoptosis in SCs.** (**A**) Representative flow cytometric plots of apoptosis assay. WI-38 non-senescent cells (NCs) and IR‐induced senescent cells (IR-SCs) were pretreated with vehicle (Veh) or 10 µM pan-caspase inhibitor Q‐VD‐Oph (QVD), and then treated with 2 µM EF24 for 72 h. Cell apoptosis was assayed by flow cytometer after Annexin-V and PI staining. (**B**) Percentage of apoptotic (PI^-^ Annexin V^+^ and PI^+^ Annexin V^+^) NCs. (**C**) Percentage of apoptotic (PI^-^ Annexin V^+^ and PI^+^ Annexin V^+^) IR‐SCs. Data are represented as mean ± SEM of three independent assays. ****P < 0.0001.

Next we determined whether EF24 reduces WI-38 IR-SCs viability through induction of apoptosis. This was done by measuring the percentage of Annexin-V positive and Annexin-V/propidium iodide (PI) double positive apoptotic cells by flow cytometry after Annexin-V and PI staining. As shown in Figure 3A and B, EF24 treatment did not induce apoptosis in NCs. However, it significantly increased the number and percentage of Annexin-V positive apoptotic cells in IR-SCs compared to vehicle treatment. To determine whether EF24 induces SCs apoptosis via activation of caspases, we next treated NCs and IR-SCs with Q-VD-OPh (QVD, a pan-caspase inhibitor) [26] prior to the addition of vehicle or EF24. The data show that QVD pretreatment completely blocked the effect of EF24 on the induction of SCs apoptosis (Figure 3A and C). However, there were not any significant changes in the number of PI positive cells representing necrotic cells in IR-SCs after EF24 treatment, suggesting EF24 does not induce IR-SCs necrosis. Collectively, these results suggest that EF24 selectively reduces SCs viability via induction of apoptosis.

### EF24 induces apoptosis in SCs in a ROS production independent manner

**Figure 4:**
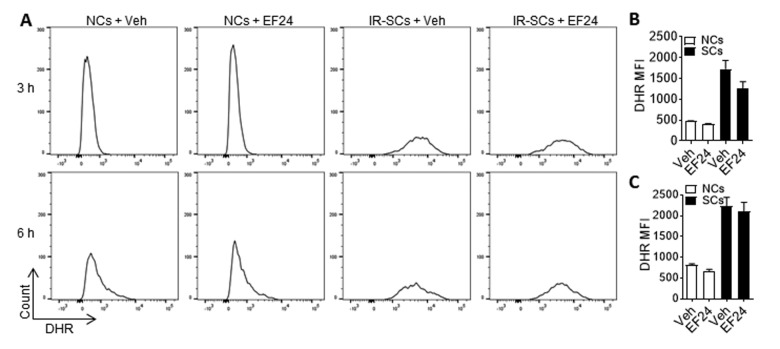
**EF24 does not increase reactive oxygen species (ROS) production.** (**A**) Representative flow cytometric plots of ROS assay. WI-38 NCs and IR‐SCs were treated with vehicle (Veh) or 2 µM EF24 for 3 h and 6 h. The levels of ROS in NCs and IR‐SCs were measured by flow cytometry after dihydrorhodamine 123 (DHR) staining. (**B**) and (**C**) ROS levels in NCs and IR-SCs after 3 h and 6 h treatment with 2 µM EF24, respectively, were presented as mean ± SEM (n = 3) of fluorescence intensity (MFI) of DHR.

It has been shown that EF24 induces apoptosis in various tumor cells in part by increasing ROS production and inducing oxidative stress-mediated endoplasmic reticulum stress [27,28]. To test whether EF24 induces SCs apoptosis also by increasing ROS production, we measured the intracellular ROS levels in WI-38 NCs and IR-SCs 3 h and 6 h after EF24 treatment using flow cytometry after dihydrorhodamine 123 (DHR) staining (Figure 4A). Compared to NCs, IR-SCs produce significantly higher levels of ROS at both time points (Figure 4B and C). Similar finding was reported by us and others before [10,29,30]. However, EF24 did not increase ROS production in NCs and IR-SCs at both 3 h and 6 h (Figure 4B and C). Even when the cells were treated with EF24 for a longer duration (i.e. for 24 h and 72 h), we still could not detect any changes in ROS production in the cells (data not shown). This finding suggests that EF24 induces apoptosis in SCs in a ROS production independent manner.

### EF24 promotes proteasome degradation of the Bcl-2 family anti-apoptotic proteins selectively in SCs

**Figure 5:**
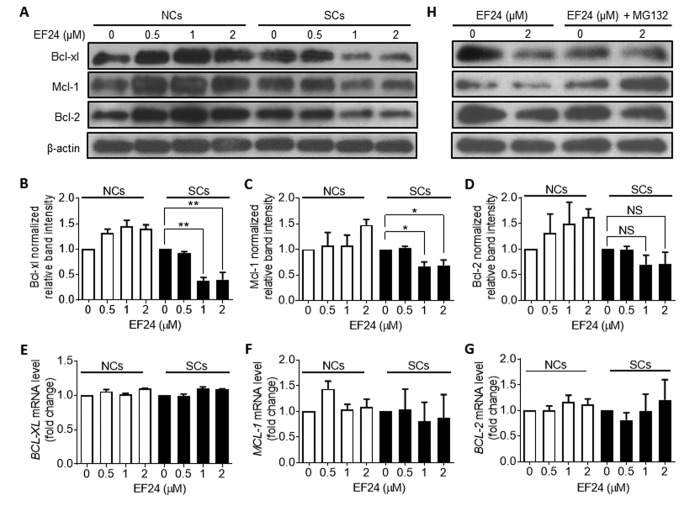
**EF24 downregulates the expression of the Bcl-2 anti-apoptotic family proteins in a proteasome-dependent manner.** (**A**) Expression of Bcl-xl, Mcl-1 and Bcl-2 in WI-38 non-senescent cells (NCs) and IR-induced senescent cells (IR-SCs) after incubation with indicated concentrations of EF24 for 72 h. Protein levels were determined by western blots, and β‐actin was used as a loading control. (**B-D**) Quantification of Bcl-xl (**B**), Mcl-1(**C**) and Bcl-2 (**D**) protein expression in WI-38 NCs and IR‐SCs after treatment with indicated concentrations of EF24 for 72 h. Data are represented as mean ± SEM of three independent assays. *P < 0.05. **P < 0.01. (**E-G**) The mRNA levels of BCL-XL (**F**), MCL-1 (**G**) and BCL-2 (**H**) in WI-38 NCs and IR‐SCs after 72 h incubation with indicated concentrations of EF24. Results were normalized as fold change in mRNA expression compared to vehicle-treated control cells. Data are represented as mean ± SEM from three independent experiments. (**H**) Proteasome inhibition with MG132 blocks the effect of EF24 on Bcl-xl, Mcl-1, and Bcl-2 expression in WI-38 NC and IR‐SC cells. Cells were pretreated with 1 µM MG132 for 1 h, followed by treatment with indicated concentrations of EF24 for 72 h.

To further explore the mechanism by which EF24 selectively induces SCs apoptosis, we examined the effect of EF24 on the expression of the Bcl-2 family anti-apoptotic proteins by western blots, because we and others previously demonstrated that Bcl-xl and Bcl-2 were upregulated in SCs to protect SCs from apoptosis [31–33]. The results show that EF24 could significantly reduce the expression of Bcl-xl and Mcl-1 in WI-38 IR-SCs but not in NCs in a dose-dependent manner (Figure 5A-C). Although the levels of Bcl-2 were also slightly reduced in IR-SCs after EF24 treatment but its reduction did not reach the statistical significance (Figure 5A and D). To test whether EF24 downregulates the Bcl-2 family proteins through regulating their gene transcription, we quantified *BCL-XL*, *MCL-1* and *BCL-2* mRNA levels after EF24 treatment, but did not observe any significant changes in their gene expression (Figure 5E-G). Next we examined whether EF24 can regulate the expression of the Bcl-2 family anti-apoptotic proteins at the level of post-transcription, particularly via proteasome degradation. We treated WI-38 IR-SCs with the proteasome inhibitor MG132 prior to EF24 treatment and then measured the expression of these Bcl-2 family proteins by western blots. As showed in Figure 5H, pretreatment of MG132 abrogated the downregulation of these Bcl-2 family proteins by EF24 in SCs, while it had no significant effect on the expression of these proteins in NCs. These results suggest that EF24 may selectively induce SCs apoptosis by promoting proteasome degradation of the Bcl-2 family proteins. The mechanisms by which EF24 can promote the degradation of these Bcl-2 family proteins selectively in SCs have yet to be determined.

### EF24 can synergistically eliminate SCs with ABT263

**Figure 6:**
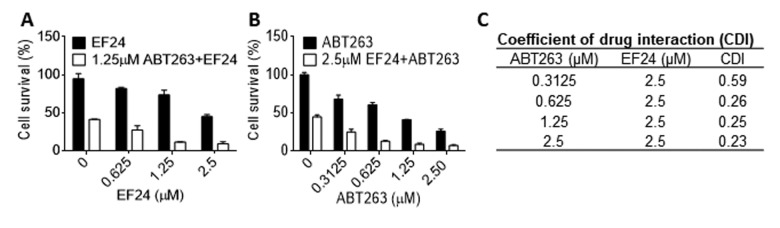
**EF24 synergistically kills SCs with ABT263.** (**A**) WI‐38 IR‐induced senescent cells (IR-SCs) were treated with indicated concentrations of EF24 in the absence or presence of 1.25 µM ABT263 for 72 h. (**B**) IR-SCs were treated with indicated concentrations of ABT263 in the absence or presence of 2.5 µM EF24 for 72 h. Cell viability was assayed by flow cytometer after PI staining. Data are represented as mean ± SEM of three independent experiments. (**C**) Coefficient of drug interaction (CDI) values were calculated for the combination treatment of 2.5 µM EF24 with indicated concentrations of ABT263.

We previously reported that ABT263 is a potent senolytic agent that can selectively kill SCs via inhibition of Bcl-xl and Bcl-2 [31]. Because EF24 can downregulate the expression of Bcl-xl in SCs, we wondered whether EF24 and ABT263 can synergistically kill SCs, which can potentially lower the dose of ABT263 needed to effectively clear SCs to reduce ABT263 toxicity. As showed in Figure 6A and B, we treated WI-38 IR-SCs with 1.25 μM ABT263 and different concentrations of EF24, or 2.5 μM EF24 with different concentrations of ABT263, and then measured the cell viability. The results from this study showed that both EF24 and ABT263 alone could dose-dependently reduce the cell viability of IR-SCs. However, the combination of ABT263 and EF24 had a synergistic effect on the reduction in SCs viability, which is confirmed by the calculation of the coefficient of drug interaction (CDI < 0.3) (Figure 6C).

## DISCUSSION

Genetically and pharmacologically selective clearance of SCs has been demonstrated to delay age-associated tissue deterioration and extend healthspan and lifespan in mice [34,35]. Hence, targeted elimination of SCs with a small molecule senolytic agent has the potential to be developed as a novel anti-aging strategy. However, most of the known senolytics except some of these natural compounds have various on- and off-target toxicities that may limit their clinical applications because old individuals are in general more vulnerable to drug toxicities than young people. For example, ABT263 has an on-target toxicity to platelets and can cause dose-limiting thrombocytopenia [36]. Treatment with HSP90 inhibitors geldanamycin and 17-AAG can lead to unacceptable hepatotoxicity [37]. Although natural compounds of senolytic agents are relatively safe, most of them suffer from low potency, cell-type specific and lack of selectivity. As an example, quercetin has been reported to be a cell-type specific senolytic agent and requires to be combined with dasatinib to effectively clear SCs in mice [8]. Flavone fisetin is also a cell type-specific senolytic agent that lacks senolytic activity against senescent human preadipocytes [9]. Therefore, to identify a more potent and broad-spectrum senolytic agent that has low drug toxicity remains an unmet task.

Curcumin, a hydrophobic polyphenol derived from the rhizome of the herb *Curcuma longa* is a well-known natural product. It has a wide-spectrum of biological and pharmacological activities [38–41]. Due to its poor drug like property and low potency, numerous curcumin analogs have been developed and exhibit an improved bioavailability and biological efficacy compared to curcumin. For example, EF24, one of the most potent curcumin analogs developed up to date, is 10-fold more potent in inducing cancer cell death than curcumin [42]. In addition, compared to the classical chemotherapy drug cisplatin, EF24 is more efficacious against various cancer cells but less toxic to normal cells [42]. The other representative curcumin analogs, including HO-3867, 2-HBA and DIMC, were also reported to have better anti-cancer activities than curcumin [43,24,25]. These curcumin analogs represent a rich resource for the discovery of novel senolytic agents because curcumin has demonstrated some anti-aging activities. In the present study, we screened several curcumin analogs and identified EF24 as a potent and broad-spectrum senolytic agent. We found that EF24 can selectively decrease the cell viability in a variety of SCs derived from various tissue origins and induced by different stresses. Its senolytic activity is likely attributable to the induction of SCs apoptosis.

EF24 was reported to selectively kill tumor cells in part by stimulating the production of ROS, resulting in collapsing of the mitochondrial membrane potential and activation of the intrinsic apoptotic signaling pathway [27,28]. However, in our study, although we noticed that SCs had a higher level of ROS compared to NCs, EF24 did not stimulate the production of ROS in SCs, indicating that EF24 uses a different mechanism to selectively kill SCs. Previous studies have shown that SCs are resistant to the induction of apoptosis due to their expression of a higher level of the Bcl-2 family anti-apoptotic proteins, such as Bcl-xl and Bcl-2 [31–33]. Targeted inhibition of these proteins with an inhibitor such as ABT263 can selectively kill SCs. In agreement with these previous findings we found that treatment of EF24 selectively reduced the expression of Bcl-xl and Mcl-1 in SCs. This reduction was not caused by the suppression of gene expression because EF24 did not change mRNA expression of the Bcl-2 family members in SCs. Instead, it may be attributable to EF24-induced increase in the degradation of these proteins by the proteasomes because proteasome inhibition with MG132 abrogated the effect of EF24. However, the mechanisms by which EF24 selectively promotes proteasome degradation of the Bcl-2 family anti-apoptotic proteins in SCs have yet to be elucidated. This unique mechanism of action of EF24 in induction of SCs apoptosis makes it suitable for the combination with an inhibitor of Bcl-2 and Bcl-xl such as ABT263 to more effectively eliminate SCs. Indeed, we found that EF24 can synergistically kill SCs when combined with ABT263. It will be of great interest to determine whether EF24 alone or in combination with ABT263 can effectively clear SCs in vivo. If the combination is more effective than either agent alone, it can potentially lower the dose of ABT263 to reduce ABT263 on-target and dose-limiting thrombocytopenia, and thus make ABT263 treatment safer for age-related diseases.

## MATERIALS AND METHODS

### Cell culture and reagents

Human WI-38 fibroblasts (WI-38, Cat. No. CCL-75), human IMR-90 fibroblasts (IMR-90, Cat. No. CCL-186), human umbilical vein endothelial cells (HUVEC, Cat. No. CRL-1730), human renal epithelial cells (HREC, Cat. No. PCS-400-012) and human pre-adipocytes (Cat. No. PCS-210-010) were newly purchased from the American Type Culture Collection (ATCC, Manassas, VA, USA). WI-38 and IMR-90 cells were cultured in complete Dulbecco’s modified Eagle medium (DMEM, Cat. No. 12430054, Thermo Fisher Scientific, Waltham, MA, USA) supplemented with 10% heat-inactivated fetal bovine serum (FBS, Cat. No. S11150H, Atlanta Biologicals, Flowery Branch, GA, USA), 100 U/mL penicillin and 100 µg/mL streptomycin (Pen Strep, Cat. No. 15140122, Thermo Fisher Scientific, Waltham, MA, USA) in a humidified incubator at 37°C and 5% CO_2_. HUVEC were cultured in EGM-2 BulletKit (Cat. No. CC-3162, Lonza, Basel, Switzerland) containing basal medium supplemented with growth factors, cytokines and other supplements according to the manufacturer’s protocol. HREC were cultured in renal epithelial cell basal medium (Cat. No. PCS-400-030, ATCC, Manassas, VA, USA) supplemented with renal epithelial cell growth kit (Cat. No. PCS400040, ATCC, Manassas, VA, USA). Human pre-adipocytes were cultured in fibroblast basal medium (Cat. No. PCS-201-030, ATCC, Manassas, VA, USA) supplemented with fibroblast growth kit-low serum (Cat. No. PCS201041, ATCC, Manassas, VA, USA). EF24 (Cat. No. E8409) and HO-3867 (Cat. No. S7501) were purchased from Sigma-Aldrich (St. Louis, MO, USA) and Selleckchem (Houston, TX, USA), respectively. DIMC (Cat. No. 10009986) and 2-HBA (Cat. No. 11879) were both purchased from Cayman Chemical (Ann Arbor, MI, USA).

### SCs induction

Three different methods were used for the induction of SCs, including replicative exhaustion, ionizing radiation and ectopic *Ras* expression as previously described [10,31]. Briefly, low-passage WI-38 cells (< 25 passages), IMR-90 cells (< 25 passages), HUVEC (< 10 passages), HREC (< 10 passages) and pre-adipocytes (< 4 passages) were used as NCs or for the induction of senescence. To induce replicative senescence (Rep-SCs), WI-38 and IMR-90 fibroblasts, HUVEC, HREC and pre-adipocytes were subcultured until they stopped to divide and became permanently growth arrested senescence after 38 passages (WI-38), 38 passages (IMR-90), 22 passages (HUVEC), 30 passages (HREC) and 10 passages (pre-adipocytes), respectively. To induce SCs by ionizing radiation (IR-SCs) and ectopic expression of *Ras* (*Ras*-SCs), WI-38 NCs were treated as previously described [10,31].

### Cell viability assay

Number of viable cells were quantified using flow cytometry as previously described with slight modifications [10,31]. Specifically, NCs and SCs were incubated with vehicle (0.1% DMSO) or a test compound at indicated concentrations for 72 h. Following dissociation with 0.25% Trypsin-EDTA (Cat. No. 25200056, Thermo Fisher Scientific, Waltham, MA, USA) at 37°C for 5 min, the cells were harvested in 100 µl pre-cooled phosphate buffered saline (PBS, Cat. No. 20012027, Thermo Fisher Scientific, Waltham, MA, USA) containing 2% FBS and 100 ng/mL propidium iodide (PI, P4170, Sigma-Aldrich, St. Louis, MO, USA) and analyzed using flow cytometry (LSR II, BD Biosciences, San Jose, CA, USA). Percentage of viable cells were determined by counting the number of PI negative cells (viable cells) and then calculated as a ratio of control cells treated with vehicle. In addition, dose-response curves were generated for each compound, and the concentration for 50% of maximal effect (EC_50_ values) was calculated using GraphPad Prism 6.

### Apoptosis analysis

WI-38 NCs and SCs were treated with vehicle or 10 µM Q-VD-OPh (QVD, Cat. No. S7311, Selleckchem, Houston, TX, USA) for 4 h prior to the addition of vehicle or 2 µM EF24 for 72 h. The suspending cells were first harvested from the culture and then pooled with the adherent cells detached by 0.25% Trypsin-EDTA at 37°C for 5 min in 12 × 75 mm polystyrene round-bottom tubes (Cat. No. 352058, Falcon, Corning, NY, USA) containing complete DMEM medium. The cells were stained with Alexa Fluor 647-Annexin V (1: 50, Cat. No. 640912, BioLegend, San Diego, CA, USA) and PI (10 µg/mL, Cat. No. 421301, BioLegend, San Diego, CA, USA) at room temperature for 30 min. The stained cells were analyzed using a BD LSR II flow cytometer.

### ROS measurement

WI-38 NCs and IR-SCs were incubated with vehicle or 2 µM EF24 for 3, 6, 24, or 72 h. After removal of suspension cells, adherent cells were detached using 0.25% Trypsin-EDTA at 37°C for 5 min. They were rinsed with DMEM once and then incubated with pre-warmed DMEM medium containing 1 µM dihydrorhodamine 123 (DHR, Cat. No. D23806, Thermo Fisher Scientific, Waltham, MA, USA) at 37°C for 30 min in dark. After washing in 4 mL DMEM with 100 ng/mL PI, cells were centrifuged immediately at 1,000 r.p.m. for 5 min to remove PI and re-suspended in DMEM medium for flow cytometry analysis. Mean fluorescence intensity (MFI) of PI negative cells was determined using the BD LSR II flow cytometer.

### Western blotting

After treatment with vehicle or EF24 at 0.5, 1 and 2 µM for 72 h, WI-38 NCs or IR-SCs were harvested in 1.5mL microcentrifuge tubes, and lysed in RIPA buffer with EDTA and EGTA (Cat. No. BP-115DG, Boston BioProducts, Ashland, MA, USA) supplemented with 1% protease inhibitor cocktail (Cat. No. P8340, Sigma-Aldrich, St. Louis, MO, USA) and 1% phosphatase inhibitor cocktail (Cat. No. P0044, Sigma-Aldrich, St. Louis, MO, USA). An equal amount of protein (30 µg/lane) from each cell extract was resolved on a precast gel (Mini-PROTEAN TGX™, Cat. No. 456-1094, Bio-Rad, Hercules, CA, USA). Proteins were blotted to a precut 0.2 µm PVDF membrane (Invitrolon™, Cat. No. LC2002, Life Technologies, Carlsbad, CA, USA) by electrophoresis. The membranes were blocked with 1X TBS-Tween (TBST, Cat. No. J77500, Affymetrix, Santan Clara, CA, USA) containing 5% non-fat dry milk (Cat. No. sc-2324, Santa Cruz Biotechnology, Dallas, TX, USA), and subsequently probed with primary antibodies at a predetermined optimal concentration overnight at 4°C. The primary antibodies including Bcl-xl (Cat. No. 2762), Mcl-1 (Cat. No. 5453), and Bcl-2 (Cat. No. 2872) and β-actin (Cat. No. 4970) were purchased from Cell Signaling Technology (Danvers, MA, USA). After washing with TBST for 3 times (10 min each time), the membranes were incubated with the secondary horse radish peroxidase (HRP)-linked antibody (Cat. No. 7074, Cell Signaling Technology, Danvers, MA, USA) for 2 h at room temperature. Following sufficient washing with TBST, the membranes were incubated with chemiluminescent HRP substrate (Immobilon™ Western, Cat. No. P36599, MilliporeSigma, Billerica, MA, USA) that consists of equal volume of luminol reagent and peroxide solution. The blotting membranes were recorded using autoradiography (SRX-101, Konica, Shinjuku, Tokyo, Japan). For the proteasome inhibition experiment, IR-SCs were treated with vehicle (0.1% DMSO) or 1 µM MG132 (Cat. No. S2619, Selleckchem, Houston, TX, USA) for 1 h prior to the incubation with EF24 at 0, 0.5, 1 and 2 µM for 72h.

### Quantitative polymerase chain reaction (qPCR)

The mRNA levels were measured with qPCR, as previously described [32]. qPCR was run with TaqMan qPCR (Cat. No. 4444965, Thermo Fisher Scientific, Waltham, MA, USA) reagents and primers (Cat. No. 4331182, Thermo Fisher Scientific, Waltham, MA, USA).

### Statistical analysis

The data were analyzed by analysis of variance (ANOVA) with GraphPad Prism 6 (GraphPad Software, La Jolla, CA, USA). In the event that ANOVA justified post hoc comparisons between group means, the comparisons were conducted using Neuman-Keuls or Tukey’s multiple-comparisons test. *P* < 0.05 was considered to be significant. To detect the synergistic senolytic activities of EF24 and ABT263, CDI was calculated as previously described [10].
